# Supplementary material for: Mortality in Four Waves of COVID-19 Is Differently Associated with Healthcare Capacities Affected by Economic Disparities
Source: Trop Med Infect Dis. 2022 Sep 10;7(9):241. doi: 10.3390/tropicalmed7090241 (PMC9506267; doi:10.3390/tropicalmed7090241)
Supplement: Supplementary file 1 [file tropicalmed-07-00241-s001.zip › tropicalmed-1850688-Supplementary Figure S1.pdf]

Tropical Medicine and Infectious Disease

MDPI

Supplementary Materials for Mortality in Four Waves of COVID-19 is Differently Associated with Healthcare Capacities Affected by Economic Disparities

Lan Yao [1], J. Carolyn Graff [2], Lotfi Aleya [3], Jiamin Ma [1,4], Yanhong Cao [5], Wei Wei [5], Shuqiu Sun [5], Cong-Yi Wang [6], Yan Jiao [1], Weikuan Gu [1,7,*], Gang Wang [4] and Dianjun Sun [5,*]

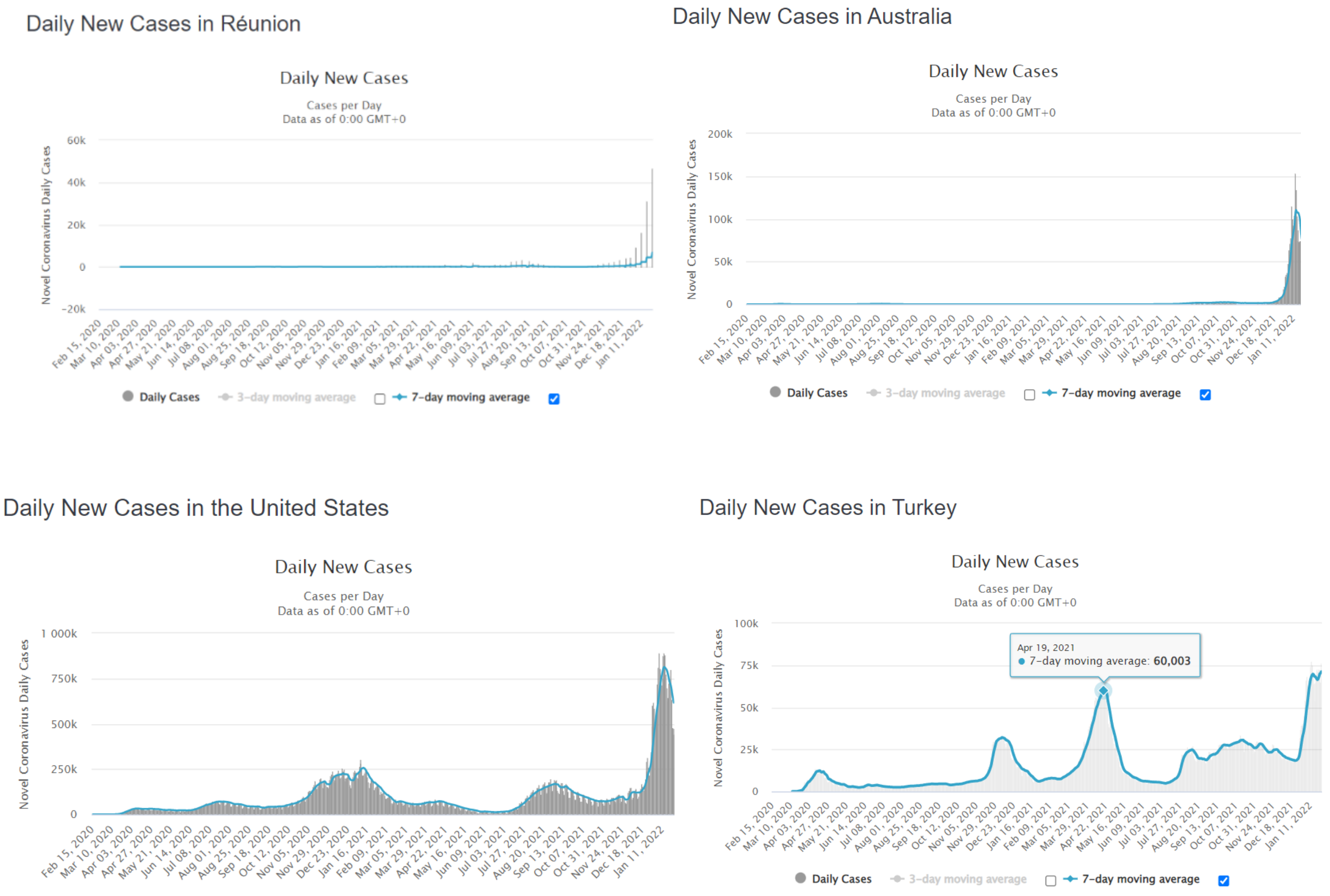

Figure S1. Example of visible and non-visible turning points.

Figure S2. Correlations of potential influential factors and death rates in four waves.
