# Supplementary material for: Mortality in Four Waves of COVID-19 Is Differently Associated with Healthcare Capacities Affected by Economic Disparities
Source: Trop Med Infect Dis. 2022 Sep 10;7(9):241. doi: 10.3390/tropicalmed7090241 (PMC9506267; doi:10.3390/tropicalmed7090241)
Supplement: Supplementary file 1 [file tropicalmed-07-00241-s001.zip › tropicalmed-1850688-Supplementary Figure S2.pdf]

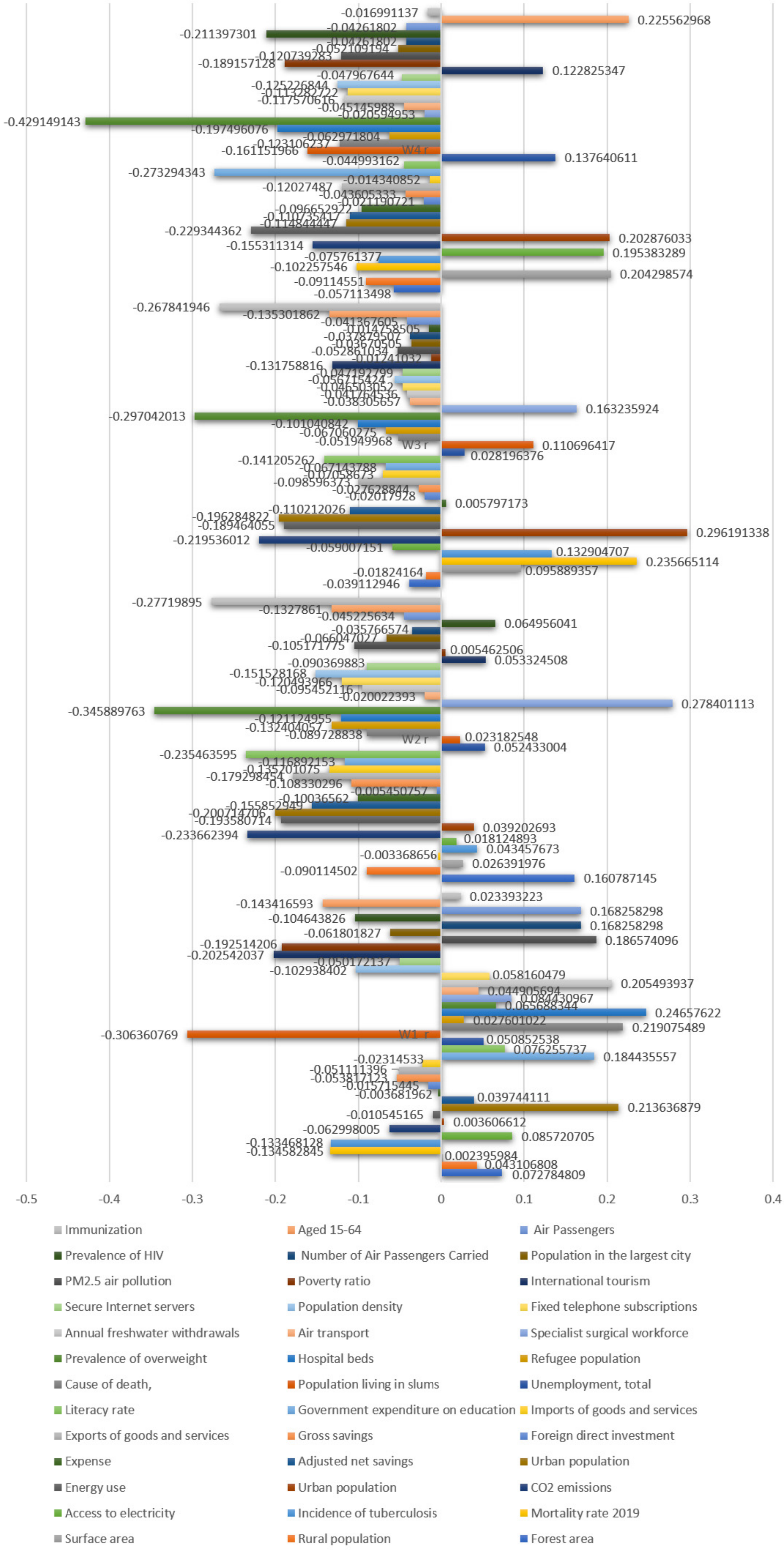

W4 r
W3 r
W2 r
W1 r
-0.5
-0.4
-0.3
-0.2
-0.1
0
0.1
0.2
0.3
0.4
Immunization
Aged 15-64
Air Passengers
Prevalence of HIV
Number of Air Passengers Carried
Population in the largest city
PM2.5 air pollution
Poverty ratio
International tourism
Secure Internet servers
Population density
Fixed telephone subscriptions
Annual freshwater withdrawals
Air transport
Specialist surgical workforce
Prevalence of overweight
Hospital beds
Refugee population
Cause of death,
Population living in slums
Unemployment, total
Literacy rate
Government expenditure on education
Imports of goods and services
Exports of goods and services
Gross savings
Foreign direct investment
Expense
Adjusted net savings
Urban population
Energy use
Urban population
CO2 emissions
Access to electricity
Incidence of tuberculosis
Mortality rate 2019
Surface area
Rural population
Forest area
